# LongGF: computational algorithm and software tool for fast and accurate detection of gene fusions by long-read transcriptome sequencing

**DOI:** 10.1186/s12864-020-07207-4

**Published:** 2020-12-29

**Authors:** Qian Liu, Yu Hu, Andres Stucky, Li Fang, Jiang F. Zhong, Kai Wang

**Affiliations:** 1 Raymond G. Perelman Center for Cellular and Molecular Therapeutics, Children’s Hospital of Philadelphia, Philadelphia, PA 19104 USA; 2 Department of Otolaryngology, Keck School of Medicine, University of Southern California, Los Angeles, CA 90033 USA; 3 Department of Pathology and Laboratory Medicine, Perelman School of Medicine, University of Pennsylvania, Philadelphia, PA 19104 USA

**Keywords:** Gene fusion, Long-read sequencing, Transcriptome sequencing, Computational tool

## Abstract

**Background:**

Long-read RNA-Seq techniques can generate reads that encompass a large proportion or the entire mRNA/cDNA molecules, so they are expected to address inherited limitations of short-read RNA-Seq techniques that typically generate < 150 bp reads. However, there is a general lack of software tools for gene fusion detection from long-read RNA-seq data, which takes into account the high basecalling error rates and the presence of alignment errors.

**Results:**

In this study, we developed a fast computational tool, LongGF, to efficiently detect candidate gene fusions from long-read RNA-seq data, including cDNA sequencing data and direct mRNA sequencing data. We evaluated LongGF on tens of simulated long-read RNA-seq datasets, and demonstrated its superior performance in gene fusion detection. We also tested LongGF on a Nanopore direct mRNA sequencing dataset and a PacBio sequencing dataset generated on a mixture of 10 cancer cell lines, and found that LongGF achieved better performance to detect known gene fusions over existing computational tools. Furthermore, we tested LongGF on a Nanopore cDNA sequencing dataset on acute myeloid leukemia, and pinpointed the exact location of a translocation (previously known in cytogenetic resolution) in base resolution, which was further validated by Sanger sequencing.

**Conclusions:**

In summary, LongGF will greatly facilitate the discovery of candidate gene fusion events from long-read RNA-Seq data, especially in cancer samples. LongGF is implemented in C++ and is available at https://github.com/WGLab/LongGF.

## Background

Gene fusion is a process by which two or more distinct genes are fused into a single gene [1]. Gene fusion could be the results of trans-splicing events or structural variants such as chromosomal translocation, interstitial deletion or chromosomal inversion. Gene fusion plays a critical role in transcriptome diversity and may be associated with human diseases, especially cancer. One of the first known gene fusions was reported to induce chronic myeloid leukemia [2], and since then, more and more gene fusions have been found to play a critical role in tumorigenesis [3–5], such as Ewing’s sarcoma and synovial sarcoma [6, 7], prostate cancer [8], breast cancer [9], bladder cancer [10], colorectal cancer [11], ovarian cancer [12], lung cancer [13] and tumors in central nervous systems [14, 15]. Importantly, gene fusions can be used as biomarkers for cancer diagnosis, such as in breast cancer [16] and ovarian cancer [17], and also used as therapeutic targets for cancer [18–21]. The ability to target and better understand gene fusions may lead to the development of novel targeted therapies in the future.

Gene fusions at a transcriptome-wide scale can be detected using RNA-seq techniques, and tens of computational methods have already been developed for this purpose on short-read RNA-seq data, including alignment-based and assembly-based approaches. Alignment-based methods detect gene-fusions on short reads mapped to annotated reference genome or transcriptome, such as Arriba [22], ChimeraScan [23], ChimPipe [24], deFuse [25], FusionCatcher [26], FusionHunter [27], FusionMap [28], FusionQ [29], FusionScan [30], InFusion [31], MapSplice [32], PRADA [33], SnowShoes-FTD [34], SOAPfuse [35], Star-fusion [36], STARChip [37], STAR-SEQR (https://github.com/ExpressionAnalysis/STAR-SEQR), and Tophat-fusion [38]. Assembly-based methods, such as BreakFusion [39], EricScript [40], Fusion-Bloom [41], FuSeq [42], JAFFA [43], NeoFuse [44], nFuse [45], Pizzly [46] and ShortFuse [47], predict gene-fusions by identifying break points using assembly sequences from short-reads. Several review studies have assessed different methods on both simulation data and real short-read RNA-seq data [36, 48, 49], and evaluated the performance in detecting gene fusions. Both alignment- and assembly-based methods require the availability of specific reads in capturing informative transcript sequence to identify fusion points. However, short-read data (typically < 150 bp) has inherited limitations to detect full length of gene isoforms, suffers from assembly ambiguity, and cannot resolve repetitive regions or low-complexity regions. Long-read RNA-seq techniques can generate sequenced reads with tens of thousands of bases, and thus can capture the majority of transcriptional isoforms in single reads without transcriptome assembly.

In the past few years, long-read RNA-Seq techniques are increasingly recognized to improve our understanding of transcriptomic complexity over short-read RNA-Seq. Computational tools designed for long-read RNA-Seq data, such as Mandalorion [50], FLAIR [51] and LIQA [52], can identify novel transcripts and quantify isoform specific expression levels. However, to our knowledge, there are limited available tools to detect gene fusions on long-read RNA-seq data. In this study, we proposed a novel approach called LongGF to detect candidate gene fusion events from long-read RNA-seq data. We examined the performance characteristics of LongGF on a set of simulation data. To further evaluate the real-world utility of LongGF, we tested LongGF on several long-read RNA-seq data sets: Oxford Nanopore data (via direct mRNA sequencing) and PacBio data on the universal human reference RNA-seq sample, as well as Nanopore data (via full-length cDNA sequencing) of a patient with acute myeloid leukemia (AML). We compared LongGF against short-read gene fusion detectors, Tophat-Fusion and STAR-Fusion, together with a hybrid method IDP-Fusion. Our evaluation demonstrated that LongGF successfully detected candidate gene fusions from long-read RNA-seq data, and some of these fusions are previously known or can be validated by additional Sanger sequencing.

## Methods

### Framework of LongGF

**Fig. 1:**
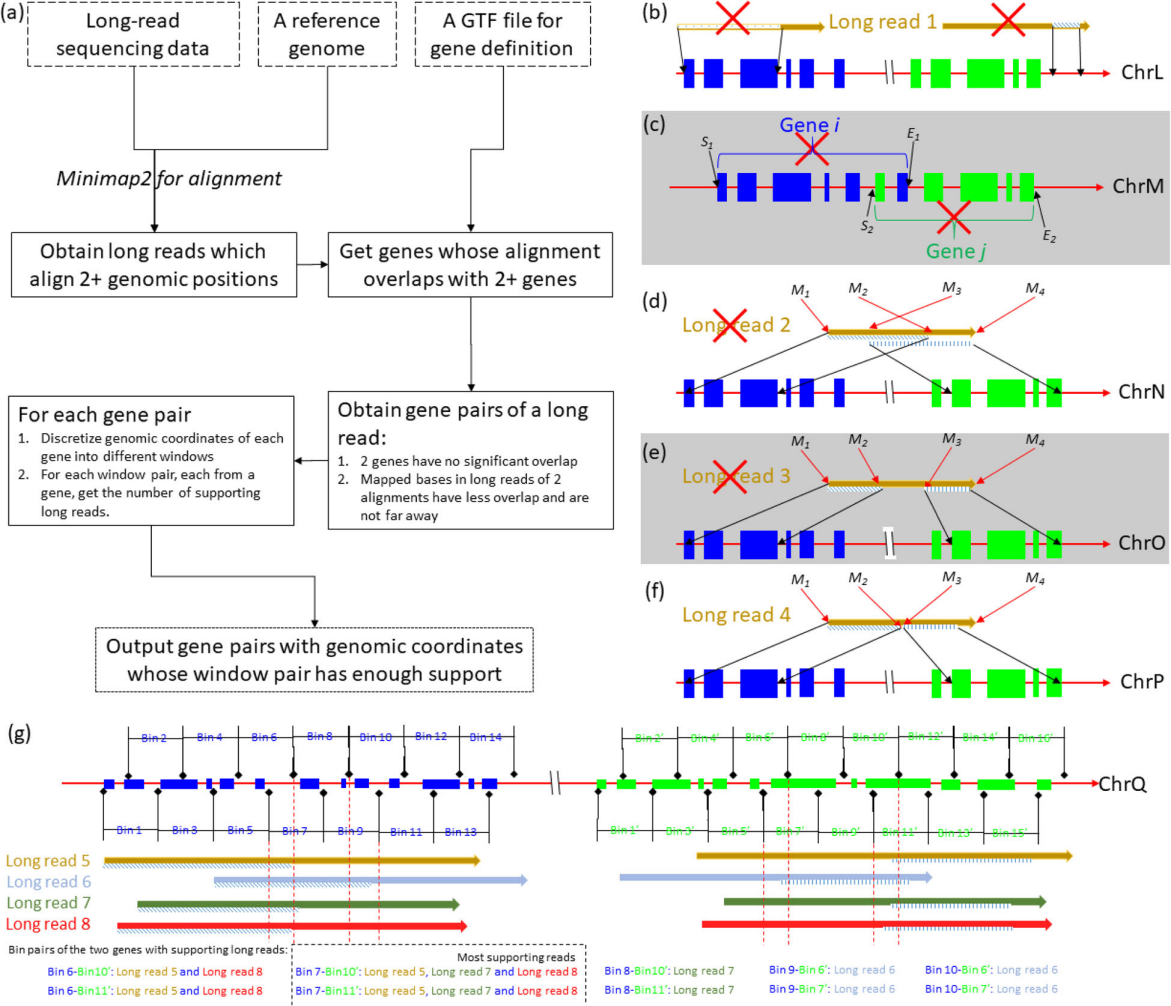
Illustration of the computational procedure used by LongGF. **a** A flowchart on LongGF, where the dashed boxes represent inputs. In panel (**b**) to (**f**), filled rectangles denote exons of gene and different colors denote different genes. The size of exons and bins are not proportional for demonstration purpose only. Pattern filled regions in long reads represent mapped bases in long reads. Information with red cross is not used for further analysis. **b** alignment against no gene; **c** two genes with significant overlap; **d** two alignments of a long read overlap significantly; **e** two alignments of a long read are far away; **f** two alignments of a long read are used for further analysis; **g** bin(window) pairs for two genes (in blue and in green) with the same aligned long reads. Dotted vertical lines help determine how alignment ends fall into bins

As shown in Fig. 1 (a), the input of LongGF is a BAM file from a long-read RNA-seq data together with a GTF file containing the definition of known genes and their transcriptional isoforms. The BAM file can be generated by different long-read aligners (minimap2 [53] by default). The output of LongGF is a prioritized list of candidate gene fusions together with their supporting long reads. LongGF has several steps to detect gene fusions from the BAM file: get multiple mapped long reads (i.e., reads that map to multiple genomic locations), obtain candidate gene pairs, find gene pairs with non-random supporting long reads, and output prioritized list of candidate gene fusions ranked by the number of supporting reads.

#### Get multiple mapped long reads

Given an input BAM file for a RNA-seq data set, we check each mapped record, and filter those long reads which have no supplementary alignment. In BAM format, a long read may have more than 1 significant alignment records in different genomic positions (as shown in Fig. 1 where both long read 1 and long read 2 have 2 alignment records), and one of them is considered as primary alignment, yet others are considered as supplementary alignments if mapped bases in the long read in this alignment have less overlap with mapped bases in the primary alignment, or as secondary alignment otherwise. Secondary alignments are thus filtered in LongGF, because upstream sequence and downstream sequence around the fusion points of gene fusion are from two independent genes and would have no substantial overlap. Thus, only primary and supplementary alignments are considered in this step.

#### Obtain candidate gene pairs of multiple mapped long reads

For each alignment record for a long read, the mapped genomic positions in a reference genome are compared against a corresponding gene definition with genomic coordinates of exon for each transcript of genes (as shown in Fig. 1 where long reads 2, 3 and 4 are mapped to different genes). If the size of the overlap of the mapped positions and the exons for a transcript is larger than a user-defined threshold, this alignment record is considered to be associated with the transcript. If a long read has more than 1 significant overlap against 2 independent genes, the gene pairs together with the alignment information are summarized; otherwise, it is filtered out as shown in Fig. 1(b). Meanwhile, to filter out noises, some genes or long reads below are not considered: (1) pseudogenes in a GTF gene definition file or two genes whose genomic coordinates have significant overlap: Assume two genes *i* and *j* whose starting positions are *S*_1_ and *S*_2_ (*S*_1_ < *S*_2_), and ending positions are *E*_1_ and *E*_2_, if *E*_1_ − *S*_2_ > 0, the two genes have significant overlap, as shown in Fig. 1 (c). Please note that we do not consider these pseudogenes in the analysis by default, and in LongGF, users can specify whether to use pseudogenes in gene fusion detection; and (2) a long read sequence whose mapped bases of two alignment records do not have an appropriate gap: for example in a long read sequence, the mapped bases from *M*_1_ to *M*_2_ are used in one alignment record, and bases from *M*_3_ to *M*_4_ are used in another alignment record, *M*_3_ > *M*_1_, if *M*_3_ − *M*_2_ is larger than a threshold (such as 20, as shown in Fig. 1 (e)) or less than − 20, as shown in Fig. 1 (d), the two alignment records do not have an appropriate gap. In Fig. 1, long reads 2 and 3 are excluded and long read 4 (Fig. 1 (f)) is used for further analysis. Please note that alignments shown in Fig. 1 (c) need users’ further investigation for potential gene fusions, while alignments such as those in Fig. 1 (d) and Fig. 1 (e) may indicate complex gene fusions or implicate the presence of potential structural variants.

#### Find gene pairs with non-random supporting long reads

Each gene pair generated above is associated with a set of long reads together with their alignment information. Those alignments may not be consistent due to alignment errors or sequencing errors. A consistent support is summarized using the process as shown in Fig. 1 (g): first, aligned genomic positions are discretized into a window with *w* bp and two adjacent windows have *w*/2 bp overlap; second, each gene pair is then associated with all possible window pairs; third, for each long read associated with this gene pair, if the fusion points of the two alignment records fall into a window of a gene and into a window of the other gene, the number of supporting long reads for this window pair is increased by 1; then, each window pair is associated with the number of supporting long reads together with the fusion points of two alignment records of long reads; fourth, for the window pair with maximum supporting long reads, the averaged genomic position of fusion points is considered as the fusion points of this potential fused gene. By default, one breakpoint is shown in the output for a gene pair, but users can specify the parameters in LongGF to output more breakpoints for each gene pair to facilitate downstream analysis to refine breakpoints.

#### Output prioritized list of candidate gene fusions

From a BAM file, multiple candidate gene fusions are detected, and each is associated with a list of a pair of two alignment records on long reads. We rank the potential fused genes according to the number of supporting long reads. More reliable gene fusion events usually have more supporting long reads. We also allow the extraction of reads in specific locations, so that users can easily examine the reads and alignments in visualization tools such as IGV, to visually validate whether the candidate fusion events are reliable.

### Datasets for evaluations

To evaluate the performance, we applied LongGF to several existing long-read RNA-seq data sets using Oxford Nanopore long-read techniques (PRJNA639366 and PRJNA40456 in NCBI Short Read Archive), including one direct mRNA sequencing data set, one full-length cDNA sequencing data set, as well as additional long-read RNA-seq data using PacBio sequencing techniques. We also simulated tens of long-read RNA-seq data sets. The description of the datasets is given below.

#### Long-read sequencing of universal human reference RNA-seq data

We analyzed two long-read datasets, using Nanopore sequencing and PacBio sequencing, for Universal Human Reference (UHR) RNA which comprises of mixed RNA molecules from a diverse set of 10 cancer cell lines with equal quantities of DNase-treated RNA from adenocarcinoma in mammary gland, hepatoblastoma in liver, adenocarcinoma in cervix, embryonal carcinoma in testis, glioblastoma in brain, melanoma, liposarcoma, histocytic lymphoma in histocyte macrophage, lymphoblastic leukemia and plasmacytoma in B lymphocyte. This reference sample from MicroArray Quality Control [17, 54, 55] project has been utilized in many studies. For example, Gao et al [56] sequenced this UHR RNA sample and treated it as reference to measure the technical variations of scRNA-seq data. Also, the qRT-PCR measurements of gene/isoform expressions from this sample were used to benchmark and optimize computational tools [57–61]. Direct mRNA sequencing protocol was used to generate Nanopore sequencing data, and we used Guppy for basecalling. In total, there are ~476,000 long reads with ~557 MB bases. We aligned the Nanopore RNA-seq data against a reference genome (hg38) using minimap2 [53], and 95% long reads (89% of total bases) were mapped, demonstrating very high sequencing and basecalling quality. Additionally, PacBio has used Iso-seq generated FLNC (full-length non-chimeric) long-read sequencing data for the UHR RNA samples [62]. In total, there are 6,775,127 long reads with 13.7 GB bases. We aligned PacBio long reads against hg38 using minimap2 [53], and 94% long reads with 95% bases were mapped. On the UHR RNA-seq data, the 6 well-known gene fusions used for our benchmarking study on short-read sequencing data include BCAS4-BCAS3, BCR-ABL1, ARFGEF2-SULF2, RPS6KB1-TMEM49(VMP1), TMPRSS2-ERG, and GAS6-RASA3.

#### Nanopore cDNA sequencing of a patient with AML (acute myeloid leukemia)

AML is a type of cancer where abnormal myeloblasts are made by bone marrow. Full-length double-stranded cDNA were generated from total RNA by 1D strand-switching RT protocol and the cDNA sample was sequenced using GridION Nanopore sequencer with Guppy basecalling. In total, there are 8,061,683 long reads with 6.6 GB bases. We aligned the data against a reference genome (hg38) using minimap2 [53], and 63% long reads (73% bases) were mapped, indicating moderate sequencing and basecalling quality. There is a gene fusion between RUNX1T1 and RUNX1 in this patient from previous cytogenetic studies, but with unknown genomic positions of the breakpoints.

#### Simulation of long-read RNA-seq data

We simulated tens of long-read RNA-seq data sets to evaluate the performance of LongGF for gene-fusion detection based on RefSeq gene annotation. To simulate a realistic dataset with known gene fusions, we used NanoSim to generate long-read RNA-seq data [63]. NanoSim simulator program captures the technology-specific features of long-read data and allows for adjustments upon improvement of Nanopore sequencing technology. The use of NanoSim facilitates the evaluation of LongGF in gene-fusion detection under a realistic setting. To simulate Nanopore RNA-seq reads using NanoSim, the human reference genome sequence (hg19, NCBI build 37) was downloaded from UCSC Genome Browser (https://genome.ucsc.edu/). We characterized parameters for NanoSim using an existing datasets generated from human reference RNA samples. We simulated 10 Nanopore RNA-seq samples (500,000 reads per sample). To make our simulated datasets more realistic, for each sample, we included 100 gene-fusions and assigned expressions to them based on gene expression distribution of a real RNA-seq dataset (expression TPM: 50 gene-fusions > 1000; 50 gene-fusions ranges from 10 to 1000). Specifically, for each gene-fusion, we first selected two isoform transcripts from two different genes and assigned fusion points randomly to cut each transcript into two parts (5′ end and 3′ end parts). Next, we combined the 5′ end and 3′ end parts from two different genes together to construct a simulated gene-fusion. The expression level of each gene-fusion was calculated based on the average expression between two genes from the UHR data set. Given annotated gene-fusions and expressions, we generated Nanopore reads using NanoSim. These simulated RNA-seq reads were then mapped to the hg19 reference human genome using minimap2 [53]. Then, we analyzed 10 samples respectively to detect gene fusion events, and compared them to the artificially created gene fusions.

## Results

### Performance on simulation datasets

**Fig. 2:**
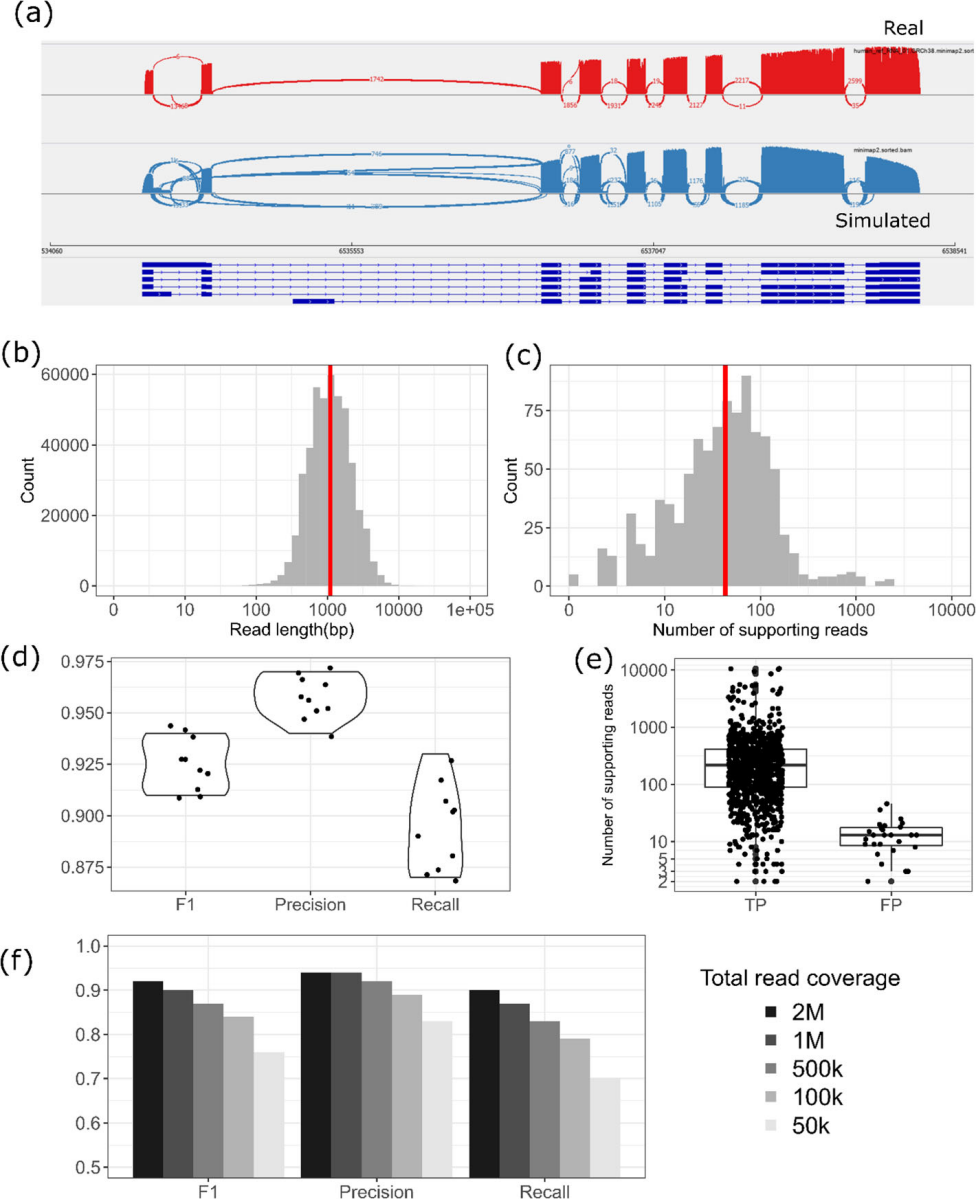
Performance evaluation of LongGF from simulation study. **a** Read coverage plots of UHR data (red) and simulated data(blue) for a random gene. **b** Read length distribution from simulated data. **c** Distribution of supporting read counts at true gene fusion point from simulated data. **d** The measurements (F1, precision and recall) of LongGF based on 10 simulations. **e** The number of supporting reads at true positive (TF) and false positive (FP). **f** The measurements (F1, precision and recall) of LongGF across different total read coverages based on the same simulation dataset

The characteristics of the simulated data are shown in Fig. 2 (a)(b)(c). The median read length is 1091 bp and the average mapping rate across 10 simulated datasets is 99%. The coverage plot of the simulated data is similar to a real study, demonstrating positional biases and full-length coverage of isoforms. For all underlying gene fusions, the median number of supporting reads is 42. Since the ground truth is known, these simulated datasets facilitate the performance evaluation of LongGF.

We explored several measures to quantify the accuracy of gene fusion detection by LongGF on simulation datasets. First, we measured the recall (power) of our method by calculating the proportion of correctly predicted gene fusions among known (artificially introduced during simulation) gene fusions. Second, we measured the precision of LongGF by calculating the proportion of correctly predicted gene fusions among all predicted gene fusions. Last, we evaluated the overall performance of LongGF using F1 score which is a weighted average of precision and recall values ($\mathrm{F}1\ \mathrm{score}=2\bullet \frac{\mathrm{precision}\bullet \mathrm{recall}}{\mathrm{precision}+\mathrm{recall}}$).

Figure 2 (d) (e) shows the distribution of supporting read counts at different events (true positive, false positive) and the summary statistics of LongGF (supporting read detection threshold ≥2) based on 10 simulations respectively. 37% (10 out of 27) of false positives have supporting read count less than 10. Clearly, LongGF has consistently high precisions (> 93%) across all simulation data sets, indicating that the false positive rate is well controlled. The recall values for LongGF range from 86 to 93%. 3 gene fusions (3%) were missed by longGF across 10 simulations on average. Most of these false negative gene fusions have low expression (TPM < 50 on average), leading to limited number of long reads that are mapped to the fusion point between genes. For example, the expression of a missed gene fusion was only 13.2 TPM, and there was no simulated read that is mapped to the fusion point. Accounting for both precision and recall values, F1 scores remain high (> 90%) for all 10 simulated datasets.

Next, we evaluated the impact of the read coverage on the accuracy of gene fusion detection. We simulated four other datasets with the fusions from the sample with the lowest F1 score (90.8%) above, and the 5 datasets for this sample have different total read coverages(2 M, 1 M, 500 k, 100 k and 50 k reads respectively). Then, we calculated recall, precision and F1 score for each dataset. As shown in Fig. 2 (f), for datasets with less number of reads, the performance becomes generally lower. Compared to the original dataset with 500 k reads, the performance measurements (recall, precision, F1 score) of 2 M reads were improved by 8.4, 2.1 and 5.7% respectively. Meanwhile, the measurements (recall, precision, F1 score) dropped by 18.5, 10.8 and 14.5% when read coverage decreased from 500 k to 50 k. This is not surprising because on lower coverage data, less number (or none) of the reads are mapped to the fusion breakpoint between two genes, which makes it more difficult for LongGF to detect candidate gene fusion events. Precision is less sensitive to the change of sequencing depth than recall. In summary, LongGF performed robustly (recall: 88.6%, precision: 95.8%, F1 score: 91.9% in average) in detecting gene fusions using simulation data with ~ 500 k reads.

### Evaluation on the UHR RNA sample by direct mRNA sequencing

**Fig. 3:**
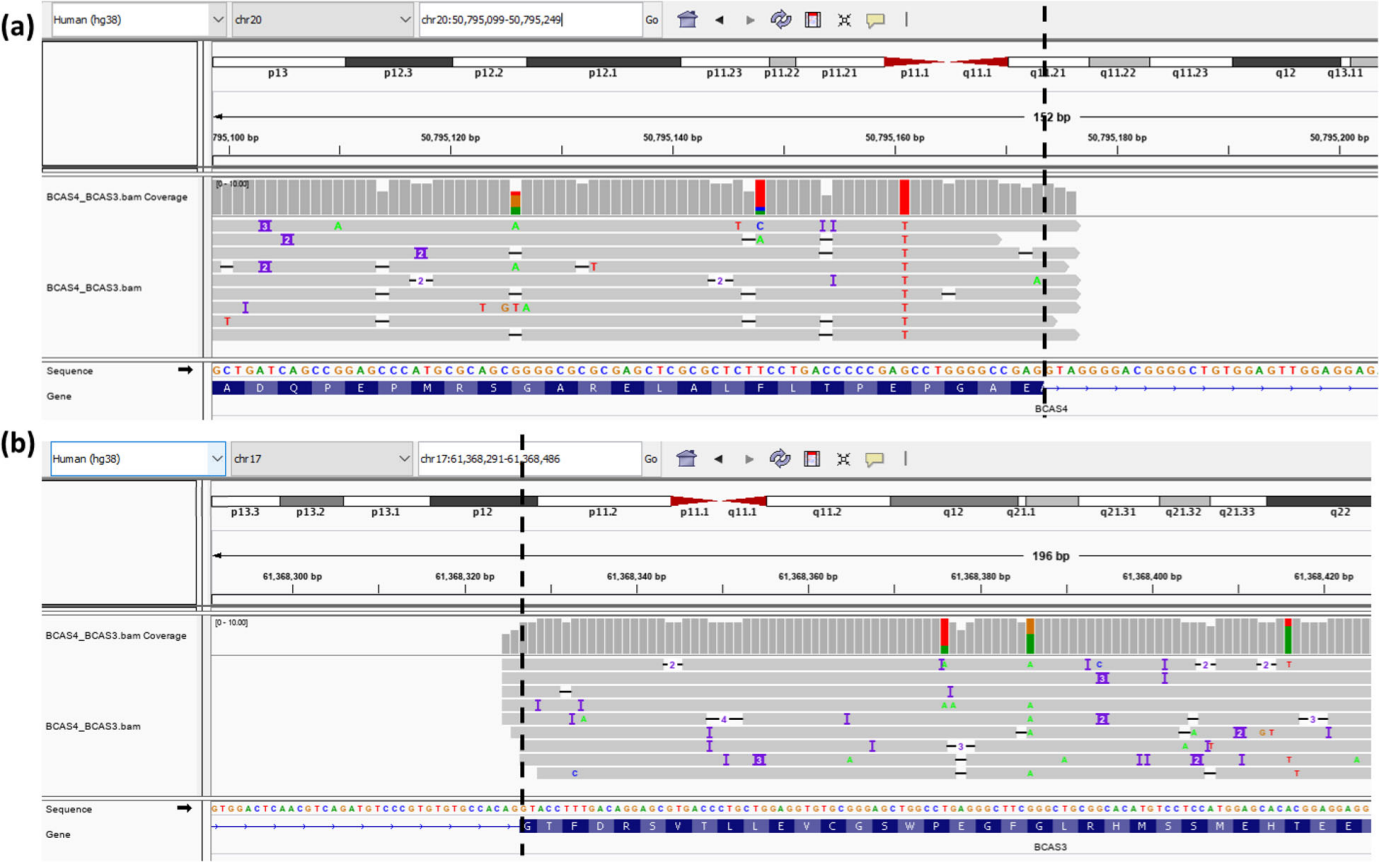
Examination of BCAS4-BCAS3 fusion in the Nanopore direct mRNA sequencing data on the UHR sample. **a** The IGV plot for the gene fusion at the genomic region around BCAS4. **b** The IGV plot for the gene fusion at the genomic region around BCAS3. The vertical dotted line indicates the genomic location where breakpoint occurs

UHR sample contains a mixture of RNA from 10 different cancers and is a widely used benchmarking material to evaluate computational tools for transcriptome analysis. We previously sequenced the sample using Oxford Nanopore direct mRNA sequencing protocols and generated ~476,000 long reads with ~ 557 M bases. After aligning the data against hg38 with minimap2 [53], we detected gene fusions using LongGF with the minimum mapped length of 100 bp, minimum overlap size of 100 bp between mapped bases and exons of a transcript and *w* = 50. The results are shown in Table 1 with the threshold of 2 for minimum supporting long reads. In Table 1, there are the 6 detected gene fusions, and 4 of them are among the 6 known gene fusions on UHR which were previously used for evaluating short-read gene fusion detectors. In particular, the top 1st gene fusion is shown in Fig. 3 in IGV plots where 9 long reads support the gene fusion very well.

**Table 1:** Candidate gene fusions detected by LongGF on long-read RNA-seq data for universal human reference mRNA sample and for a patient with AML. “A:B” denotes a gene fusion of gene A and gene B. The 6 known gene fusions for ‘UHR Nanopore’ rows are used for evaluating gene fusion detection, but additional gene fusions may be present for UHR samples

Gene fusion	Datasets	#Supporting reads	Read coverage	Fusion points (hg38 coordinate)		Benchmark
				Breakpoint 1	Breakpoint 2	
**BCAS4:BCAS3**	UHR Nanopore	8	23	chr20:50,795,173	chr17:61,368,325	Yes
MGAT5:IGLC7		7	12	chr2:134,120,290	chr22:22,922,718	
**GAS6:RASA3**		2	8	chr13:11,3826,995	chr13:113,981,855	Yes
**ARFGEF2:SULF2**		2	5	chr20:48,922,009	chr20:47,736,942	Yes
AP3D1:JSRP1		2	41	chr19:2,127,153	chr19:2,252,480	
**VMP1:RPS6KB1**		2	78	chr17:59,838,294	chr17:59,910,610	Yes
**BCAS4:BCAS3**	UHR PacBio	206	520	chr20:50795172	chr17:61368324	Yes
**ARFGEF2:SULF2**		70	389	chr20:48922011	chr20:47736941	Yes
FGFR1:NSD3		42	53	chr8:38457533	chr8:38381797	In [64]
LDLR:ZNF333		36	51	chr19:11108452	chr19:14701590	In [64]
SMARCA4:CARM1		28	1126	chr19:10986591	chr19:10904949	In [64]
**VMP1:RPS6KB1**		26	2601	chr17:59838295	chr17:59910609	Yes
**GAS6:RASA3**		22	103	chr13:113826995	chr13:113981856	Yes
GANAB:B3GAT3		20	33	chr11:62627352	chr11:62620496	In [65]
RPS6KB1:DIAPH3		19	2738	chr17:59930173	chr13:59666845	In [64]
NUP214:XKR3		19	39	chr9:131199013	chr22:16808083	In [64]
MYH6:HOMEZ		15	27	chr14:23386622	chr14:23285911	In [38]
PAPOLA:AK7		15	78	chr14:96502599	chr14:96437835	In [64]
CBX3:CCDC32		14	26	chr7:26201744	chr15:40561981	In [64]
MYH9:EIF3D		14	65	chr22:36387806	chr22:36526134	In [64]
DCAF6:SEMA4A		14	885	chr1:167951860	chr1:156153370	
ZBTB45:UBE2M		11	35	chr19:58518739	chr19:58557605	
RSBN1:AP4B1		10	15	chr1:113811708	chr1:113899869	
GCN1:MSI1		10	65	chr12:120190297	chr12:120347515	In [64]
ESR1:CCDC170		9	29	chr6:151702003	chr6:151573171	In [64]
NUP210L:GATAD2B		8	501	chr1:154027597	chr1:153922729	In [64]
**ABL1:BCR**		8	298	chr9:130854065	chr22:23290408	Yes
ZFP64:ATP1A1		8	18	chr20:52052134	chr1:116396734	
GOPC:ROS1		8	13	chr6:117566853	chr6:117321395	In [64]
RUNX1T1:RUNX1	A patient with AML	9	2373	chr8:92,017,366	chr21:34,859,474	Yes
NBEAL1:RPL12		8	7244	chr2:203,190,790	chr9:127,451,392

Note that it is likely that more than 6 true gene fusions are present in the UHR sample which is composed of 10 different cancers, but we only used the 6 well known fusions for evaluation of LongGF on UHR Nanopore data as the short-read gene fusion detectors did. That is, 2 gene fusions detected by LongGF (possible false positives) are not in the known gene fusion list, and one of them is mapped against a reference region which appears more than 1 times, and thus, this may represent a genuine gene fusion event that was missed by previous studies. Meanwhile, since 2 false negative events, including the well known BCR-ABL1 gene fusion, are not detected by us, we further examined the sequence data on the genomic region of the BCR-ABL1 gene fusion. We found that the failure to identify BCR-ABL1 gene fusion may be due to the low expression of this fusion in the transcriptome: as shown in short-read RNA-seq data of UHR [38], BCR-ABL1 is ~ 6 times less expressed than BCAS4-BCAS3 gene fusion (with 9 supporting long reads in UHR Nanopore RNA-seq data), and ~ 4 times less expressed than GAS6-RASA3 (with 2 supporting long reads in UHR Nanopore RNA-seq data) and ARFGEF2-SULF2 gene fusion (with 2 supporting long reads in UHR Nanopore RNA-seq data). Given that the UHR sample is a mixture of 10 different cancer cell lines, it is expected that known gene fusions such as BCR-ABL1 in one cell line will have relatively low allele fraction in the data; therefore, in the long-read RNA-seq data generated by us, we do not have enough coverage on the BCR-ABL1 gene fusion and we were not able to detect this fusion by LongGF.

We further evaluated LongGF on a higher coverage PacBio long-read data on the UHR sample that was sequenced by PacBio [62]. The results were shown in Table 1. It can be seen from Table 1 that LongGF is able to detect the BCR-ABL1 gene fusion and other known gene fusions detected on the Nanopore data. In particular, the BCR-ABL1 gene fusion only has 8 supporting long reads compared against other known gene fusions (206, 69, 26 and 22 supporting reads for BCAS4-BCAS3, ARFGEF2-SULF2, VMP1-RPS6KB1 and GAS6-RASA3, respectively), which supports our speculation that the low-coverage issue is the reason why LongGF on Nanopore data missed this gene fusion. Additionally, LongGF on the PacBio long-read data detects 23 gene fusions with > = 8 supporting long reads. We thus checked other detected gene fusions using Mitelman databases (Mitelman databases contains many gene fusions in cancers manually culled from the literature) [64] and other online resources [65]. We found that 19 gene fusions were reported in the literature. In contrast to short-read data, only 3 of top 20 detected gene fusions by STAR-Fusion [36] and 6 of top 20 detected gene fusions by Tophat-Fusion [38] were reported in the literature. Although this is not a direct comparison of the tools to detect gene fusions on long-read and short-read datasets, this analysis suggests that LongGF on long-read high-coverage data likely identify more reliable gene fusions with much less false positives.

### Evaluation on a breast cancer dataset

Moreover, we compared LongGF with IDP-Fusion [66], a gene fusion detector using hybrid data (both long-read and short-read sequencing data), on the MCF-7 breast cancer dataset. Among a set of 71 fusion gene events validated by either PCR and/or Sanger sequencing [66], LongGF and IDP-Fusion detected 25 and 24 events, respectively. The recall of LongGF in detecting fusion genes is comparable to IDP-Fusion on this long-read data on breast cancer, but IDP-Fusion uses both long-read and short-read data. LongGF also detected more potential novel gene fusions (ACTB:H3F3B, SLC25A24:NBPF6, STMN1:ACTG1), and these genes were reported to be associated with breast cancer [67–71]. Therefore, compared to hybrid-based gene fusion detector, LongGF yields comparable accuracy for fusion gene detection.

### Evaluation on a patient with AML by long-read cDNA sequencing

**Fig. 4:**
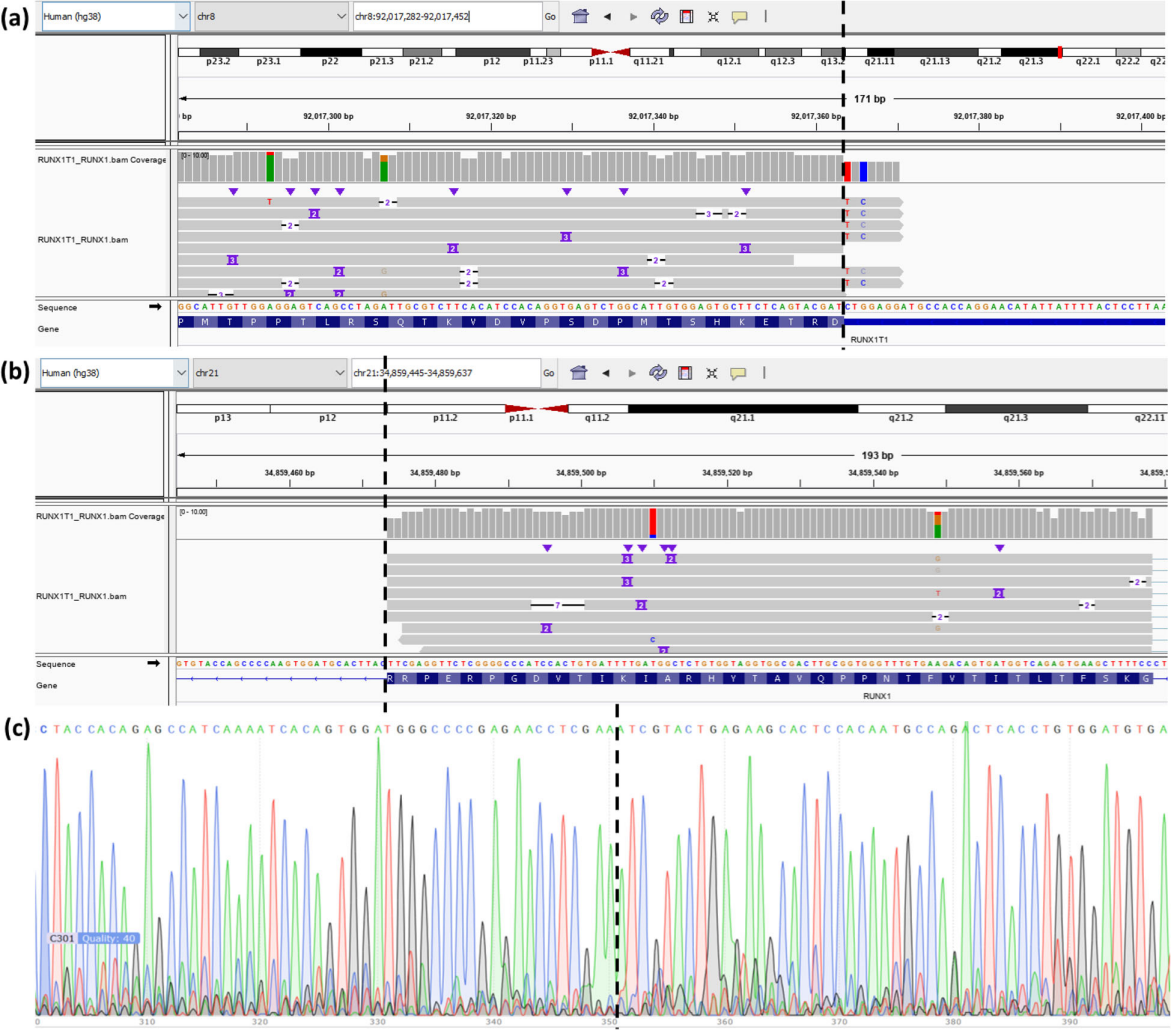
Examination of RUNX1T1-RUNX1 fusion in Nanopore long-read RNA-Seq data and Sanger sequencing data on a patient with AML. **a** The IGV plot for the gene fusion at the genomic region around RUNX1T1. **b** The IGV plot for the gene fusion at the genomic region around RUNX1. **c** the result from Sanger sequencing. The vertical dotted line indicates the genomic location where breakpoint occurs. The Sanger sequence in (**c**) is the complementary of the sequences in (**a**) and (**b**)

To further evaluate the performance of LongGF on real datasets, we analyzed a long-read cDNA sequencing data generated on blood sample from a cancer patient affected with AML. We detected gene fusions using LongGF on this long-read RNA-seq data with the minimum mapped length of 500 bp, minimum overlap size of 100 bp between mapped bases and exons of a transcript, and *w* = 50. The results are shown in Table 1 with minimum supporting reads of 5, where 2 gene fusions are detected. One detected gene fusion is *RUNX1T1*:*RUNX1* (as shown in Fig. 4 (a) and (b)), which has been found to be associated with AML [72]. We note that although the same gene fusion was previously known by cytogenetic analysis on this patient, the exact breakpoint location is not known for this patient. We then validated this gene fusion using Sanger sequencing (as shown in Fig. 4 (c)), and we found that the breakpoints of the two genes are chr8:92,017,373 and chr21:34,859,474, respectively. Compared with the results reported by LongGF, the inferred breakpoint at chr21 is exactly the same as the Sanger sequencing result, yet the inferred breakpoints at chr8 is only 6 bp away from Sanger sequencing result. Altogether, our analysis demonstrated that LongGF can detect gene fusions and infer relatively precise breakpoints using long-read RNA-seq data.

## Discussion

Gene fusion is a well-known strategy used by cells to generate new genes in transcriptome, and many existing studies have found that some gene fusions contribute to the initiation or progression of different human cancer. Although short-read RNA-seq techniques provide a way to detect gene fusions for transcriptome data, long-read RNA-seq techniques represent theoretically better solutions to overcome the limitations of short-read techniques. However, it is not straightforward to detect gene fusions from noisy long-read RNA-seq data, and in this study, we propose LongGF to detect gene fusions from long-read RNA-seq data efficiently and effectively. LongGF is implemented in C++ and is very fast to run, and it only takes several minutes and < 3GB memory on 50,000 long reads from a transcriptome for gene fusion detection. Our evaluation also showed that LongGF can accurately detect true gene fusions on simulation datasets and four real datasets. Thus, LongGF is a useful tool for long-read RNA-seq data analysis, especially on cancer samples.

However, there are some limitations in LongGF. First, LongGF cannot detect gene fusions with unknown genes, since LongGF requires a pre-defined definition of all genes/exons in a GTF file. Therefore, this version of LongGF only detects candidate gene fusions from two known genes. To allow the detection of gene fusions involving novel genes/exons, users can modify the standard GTF file and include additional genes/exons. Second, LongGF may generate false positive predictions on gene fusions when dealing with homologous genes in the genome. That is, if several genes in a transcriptome share similar sequences (possibly part of the transcript sequence), it will be difficult to distinguish which gene the fused gene comes from. For example, if gene A and gene B have similar sequence and part of gene A is fused with part of gene C for a hybrid gene D, it is hard to find whether the gene D is formed from gene A and gene C, or gene B and gene C. Third, LongGF may miss gene fusions from very short genes. In LongGF, we require an alignment is long enough to be significant and that an alignment has substantial overlap with a gene for further analysis. If only a smaller fraction (< 100 bp) of a gene is involved in a gene fusion, it is hard to distinguish the fusion candidates from sequencing/alignment noises. In LongGF, although users can set smaller thresholds to get gene fusions with smaller segments, they will generate more candidate fusion events and need to filter noisy candidates in the results.

With full-length transcriptome sequencing, we expect that long-read RNA-seq data (Oxford Nanopore and PacBio) will greatly facilitate gene fusion detection by overcoming many technical limitations of short reads. Compared to PacBio (either with traditional library or HiFi library preparation protocols), at fixed cost, Oxford Nanopore may be a more promising platform in gene fusion detection while generating data with higher error rate. This is because Nanopore currently has lower per-base cost of data generation, and our real data analysis showed that sequencing data with high read coverage can improve detection accuracy significantly. For Nanopore RNA-seq, there are two types: direct mRNA sequencing and cDNA sequencing. Compared to direct mRNA sequencing, cDNA sequencing allows samples to be amplified and requires less amount of starting materials, making it attractive in some cases. With more materials for sequencing (possibly in multiple flow cells), this can increase the read coverage at fusion breakpoint, and facilitate LongGF to detect gene fusions with low expression or low allele fraction (such as the BCR-ABL1 fusion discussed earlier). Additionally, we will conduct more comparison of the performance between LongGF with existing short read tools, for samples where both short-read and long-read sequencing data are available and the sample is available for experimental validation. We expect that we may find fusion events that are missed by short-read sequencing approaches, even when the sequencing coverage in short-read data is very high, if part of the fusion event falls under repetitive genomic regions.

## Conclusion

In summary, LongGF is a fast and effective computational tool to detect candidate gene fusions from long-read RNA-seq data. With the advancement of long-read sequencing techniques, we expect that LongGF will significantly contribute to the discovery of disease-causal gene fusions in the studies of human genetic diseases and cancer.

## Data Availability

The direct mRNA sequencing data on UHR and cDNA sequencing data on AML are available at NCBI Short Read Archive under BioProject accession PRJNA639366 and PRJNA640456, respectively. The LongGF software tool is available at https://github.com/WGLab/LongGF.

## Abbreviations

LongGF: Long-read gene fusion detector
UHR: Universal Human Reference RNA sample
AML: Acute myeloid leukemia
